# Ramadan fasting and type 1 diabetes: on a case successfully managed with an integrated system based on predictive low-glucose suspend algorithm

**DOI:** 10.1007/s00592-021-01693-y

**Published:** 2021-03-08

**Authors:** Marina Valenzano, Elena Gamarra, Giorgio Grassi

**Affiliations:** 1 Division of Endocrinology, Diabetes and Metabolic Diseases, Department of Medical Sciences, University of Turin, A.O.U. Città della Salute e della Scienza – Le Molinette Hospital, Corso Bramante 88, 10126 Turin, Italy; 2 Present Address: Clinique Et Centre Médical Thérapeutique La Lignière, 1196 Gland, Switzerland

**Keywords:** Type 1 Diabetes, Ramadan, Fasting, Predictive low-glucose suspend algorithm, Pump therapy, Integrated systems for insulin delivery

## Introduction

The number of Muslim people with diabetes is expected to increase also in Western Countries [1]. Performing Ramadan fasting is a strong desire for many Muslim patients despite diabetes; in fact, a large epidemiological study [2] in the early 2000s confirmed that a consistent number of them, both with type 1 and type 2 diabetes, try to fast for several days, often without proper medical supervision, which may lead to complications like hypoglycaemia, hyperglycaemia, ketoacidosis, dehydration and thrombosis. International guidelines [1] provide healthcare professionals with the information needed to select patients who should avoid fasting because of excessive health risk and to support those who persist with their decision to fast. However, scarce evidence is available regarding the application of new technologies for diabetes care in this specific subset. A recent systematic review and meta-analysis about safety of Ramadan fasting in type 1 diabetes [3] suggests that fine-tuning of insulin therapy by the use of pump infusion and intensive glucose control may mitigate hypoglycaemia risk. Further, an observational study has compared sensor-augmented pump therapy (SAP), equipped with a low-glucose suspend (LGS) function, to traditional continuous insulin infusion in association with continuous glucose monitoring (CGM) and no automatic suspension [4]. Although the SAP system mentioned above proved to be safe and to reduce both hypo- and hyper-glycaemic excursions, it has now been replaced by more advanced models and is no longer commercialised. Finally, there is no consensus regarding manual insulin dose adjustments, while substantial evidence is still lacking on the use of automatic insulin delivery to the specific setting of Ramadan, except for a single report on a hybrid closed-loop system.

We here report the case of a patient with type 1 diabetes who, because of several severe hypoglycaemic episodes, had previously failed to fast during Ramadan and who volunteered to pursue a new attempt using advanced technologies for diabetes care.

## Case history

A 20-year-old young Italian woman of Egyptian origin (body mass index 25 kg/m^2^) wished to fast during Ramadan. She was affected by type 1 diabetes since the age of 3, treated with multiple daily insulin injections. The patient showed no chronic complications and no comorbidities, except for autoimmune thyroid disease with no need for replacement therapy. Her glucose control had always been close to target (HbA1c 53–59 mmol/mol, 7–7.5%), with no episodes of ketoacidosis or severe hypoglycaemia. Latest daily insulin requirement was 61 IU (e.g. 0.9 IU/kg/die), consisting of 41% basal insulin and 59% boluses. The patient was instructed about carbohydrate counting. As mentioned, the patient had previously unsuccessfully attempted to fast during Ramadan but several hypoglycaemic episodes forced her to interrupt. During the last attempt, she experienced worsening of metabolic control (HbA1c up to 75 mmol/mol, 9.0%). The patient was willing to use diabetes treatment technologies to try Ramadan fasting again.

## Intervention

In February 2020, the patient started pump therapy with integrated continuous glucose monitoring.

The integrated system consisted of the Basal-IQ® technology on the t:slim X2™ pump (Tandem Diabetes Care Inc., San Diego, California), based on a predictive low-glucose suspend algorithm (PLGS). Thanks to the PLGS function, insulin delivery is temporarily blocked whenever sensor-glucose is expected to become lower than 80 mg/dl (4.4 mmol/L) in the following 30 min. Interruption of delivery may last for a maximum of 2 h in a 2.5-h window and is automatically resumed as soon as glucose trend reverts.

Then, according to the IDF-DAR [1] and Italian guidelines, the patient was assigned to a moderate risk category and therefore allowed to fast. In April 2020, she received special education regarding diet counselling and self-monitoring of glucose. Her pump was set up with a tailored basal insulin profile: insulin basal rate was reduced by 38% in the last 4 h of fasting (corresponding to a 14% reduction in morning daily dose), as recommended in [1]. The nocturnal settings, insulin sensitivity factor and insulin/carbohydrates (I/CHO) ratios were left unchanged. To further decrease the risk of hypoglycaemia due to aggressive corrections, optimal glycaemic target for bolus calculator was increased by 12.5%.

## Outcome

**Fig.1:**
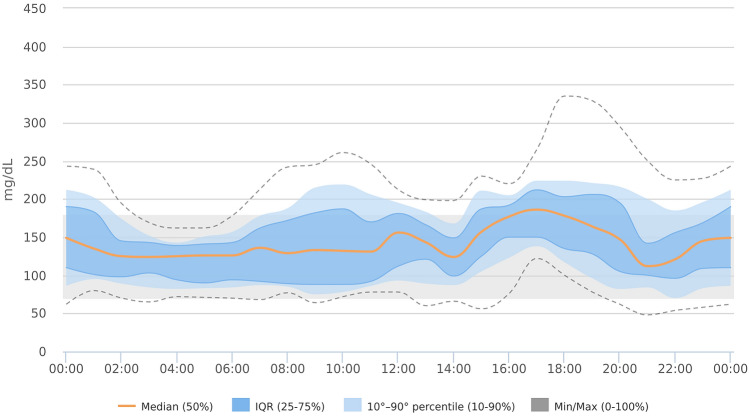
Ambulatory glucose profile showing 24-h glucose overlay of the last 14 days before Ramadan fasting (graphical elaboration by Diasend-Glooko®, Glooko Inc, Palo Alto, California)

**Fig.2:**
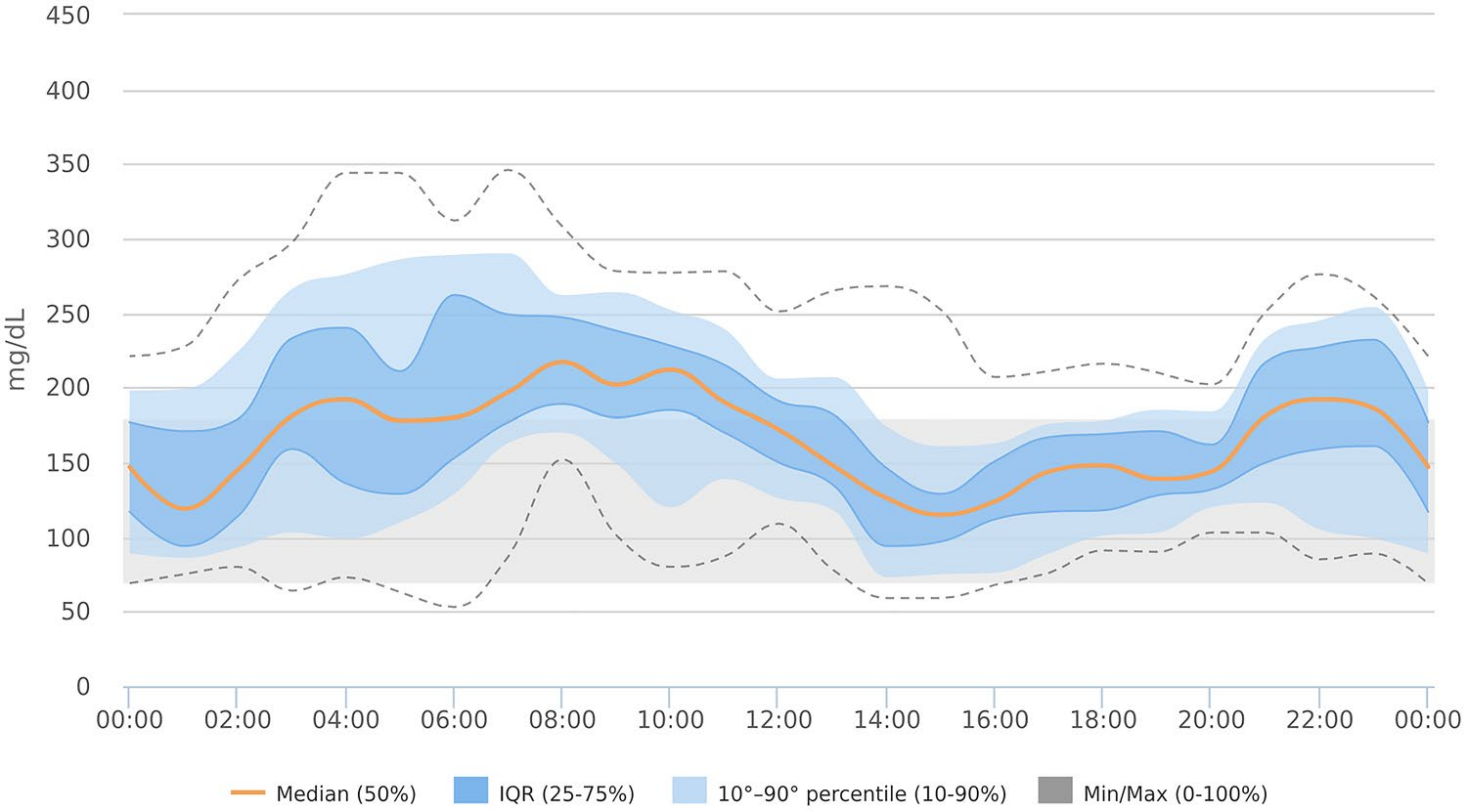
Ambulatory glucose profile showing 24-h glucose overlay during Ramadan (graphical elaboration by Diasend-Glooko®, Glooko Inc, Palo Alto, California)

The patient achieved optimal glucose control before Ramadan: mean glucose (MG) 145 + 45 mg/dl (8.1 + 2.5 mmol/L) (mean + SD), coefficient of glucose variability (CV) 32%, time in target range 70–180 mg/dl (3.9–10.0 mmol/L) (TIR) 78%, time below target range (TBR) 2%, time above target range (TAR) 20%, glucose management indicator (GMI) 49.7 mmol/mol or 6.7%. The last 14-day ambulatory glucose profile is shown in Fig. 1. The patient started fasting on April 24th 2020 and stopped on May 14th, exempted because of her menstrual cycle. The patient was allowed to eat dates and typical Egyptian food during Iftar and Suhoor (meals taken after sunset and before dawn, respectively) and practised some physical exercise between the two meals, with a daily carbohydrate intake of 179 ± 59 g. Notably, the patient never had to interrupt daytime fasting because of low glucose values. At the end of her Ramadan fasting, glucose control was still in safe ranges: MG 170 ± 54 mg/dl (9.4 + 3.0 mmol/L), CV 32%, TIR 60%, TBR 1%, TAR 39%, GMI 58.5 mmol/mol or 7.5%. The ambulatory glucose profile of the whole fasting period is shown in Fig. 2. Low glucose values were infrequent and clinically mild (never lower than 54 mg/dl (3.0 mmol/L) or requiring assistance from bystanders). The predictive algorithm worked through targeted insulin suspensions: 3.7 + 2.8 suspension events/day, corresponding to 61 ± 42 min/day, preferentially occurring during afternoon and early evening (27% suspension events occurred in only 4 h, from 2:00 to 6:00 pm). Although basal rates and the I/CHO ratios were progressively tuned according to the physician’s advice, a tendency towards hyperglycaemia was observed especially after both Iftar and Suhoor meals, with mean glucose values 187 + 49 mg/dl (10.4 + 2.7 mmol/L) from 10:00 to 12:00 pm, and 209 + 50 mg/dl (11.6 + 2.8 mmol/L) from 6:00 to 10:00 am. Ketones in capillary blood were self-measured and found in the low-normal range. Finally, during Ramadan, a 40% reduction in daily insulin dose was observed with a total need of 37 IU (e.g. 0.55 IU/kg), 54% basal insulin and 46% boluses. In fact, such a continuous therapy modulation led to an extra basal rate decrease in the morning hours, with an overall 25% reduction in the pre-Ramadan dose. Conversely, nocturnal basal rates increased by 13%, with the most significant modification concerning the first 3 h following Iftar (36% increase) and 2 h after Suhoor (10% increase).

The patient’s satisfaction was assessed at the end of the fasting period by the eight item-based Diabetes Treatment Satisfaction Questionnaire (DTSQ) (score range 1–6 for each item for a total range of 8–48) [5]. The score was 34, well above the expected value of 21, higher scores referring to treatment flexibility, expanded knowledge of diabetes and propensity to recommend the same treatment to other patients with diabetes willing to observe Ramadan fasting. Overall, the patient defined herself gratified and self-confident.

## Discussion

Managing the Ramadan fasting is challenging for insulin-treated patients. New technologies help address this problem, although the quick turn over of devices with advancement of functions and the small body of available evidence have not allowed the editing of full guidance, so far. We here present the case of a young woman with type 1 diabetes with a previous failure of Ramadan fasting who pursued a new attempt using a device based on PLGS algorithm. Self-management of disease was satisfactory before fasting and still in safe ranges at the end of the experience, with the patient feeling overall gratified.

Compared to a previous work [4], we observed similar results in terms of low-glucose episodes and insulin suspension process, although a different device (with a PLGS algorithm instead of LGS), which limited the patient’s workload implying neither suspension alerts nor requests for sensor-calibrations, was used.

Insulin doses were initially adjusted according to international guidelines but required further modulation, finally leading to a therapeutic programme that was closer to previously suggested settings for the use of different technology [4]. Conversely, an inversion of the basal/boluses proportion was observed as a possible consequence of the prudent strategy adopted, both by the patient and by the physician, aimed at preventing hypoglycaemia. It is likely that this approach also underlies some of the modest worsening of nocturnal hyperglycaemia and eventually of the HbA1c value, shown by our patient.

In all, we believe that our experience delivers further confidence in the use of automated technology provided that careful medical supervision is guaranteed alongside timely and informed patient interventions.

## Conclusions

Since prolonged fasting and sudden shift in mealtimes may lead to acute complications, management of glucose control during Ramadan is challenging and requires caution. The experience we report here suggests that, in well-instructed and strongly motivated patients observing Ramadan fasting, technology may greatly limit the risk of hypoglycaemia, while helping to achieve fair glucose control, reduce insulin doses and provide empowerment and satisfaction. It is conceivable that progress in diabetes treatment technologies will soon allow even more flexible and personalised therapeutic approaches, thus making Ramadan fasting more easily feasible for people with diabetes.

## Data Availability

Not applicable.
